# Automatic differentiation of Parkinson’s disease motor subtypes based on deep learning and radiomics

**DOI:** 10.3389/fneur.2025.1650985

**Published:** 2025-09-04

**Authors:** Dongming Hui, Xia Wang, Lu Xie, Fengxi Chen, Yi Guo, Yaxi Luo, Xiaojing He

**Affiliations:** ^1^Department of Radiology, The Second Affiliated Hospital of Chongqing Medical University, Chongqing, China; ^2^Department of Radiology, Chongqing Western Hospital, Chongqing, China; ^3^Medical Technology, Community Health Center of Yubei in Sha Ping Ba, Chongqing, China; ^4^7T Magnetic Resonance Translational Medicine Research Center, Department of Radiology, Southwest Hospital, Army Medical University (Third Military Medical University), Chongqing, China; ^5^Medical Imaging Department, Chongqing University Central Hospital (The Fourth People’s Hospital of Chongqing), Chongqing, China; ^6^Department of Radiology, Renji Hospital, School of Medicine, Chongqing University (The Fifth People’s Hospital of Chongqing), Chongqing, China

**Keywords:** Parkinson’s disease, motor subtypes, deep learning, radiomics, magnetic resonance imaging

## Abstract

**Objective:**

Parkinson’s disease (PD) is a common neurodegenerative disorder, and the early and accurate differentiation of its motor subtypes is of significant importance for clinical diagnosis and treatment planning. Research has shown that deep brain nuclei such as the thalamus, caudate nucleus, putamen, and globus pallidus play a critical role in the pathogenesis of different motor subtypes of Parkinson’s disease. This study aims to utilize deep learning and radiomics technology to establish an automated method for differentiating motor subtypes of Parkinson’s disease.

**Methods:**

The data for this study were obtained from the Parkinson’s Progression Markers Initiative (PPMI) database, including a total of 135 Parkinson’s disease patients, comprising 43 cases of the Postural Instability/Gait Difficulty (PIGD) subtype and 92 cases of the Tremor Dominant (TD) subtype. High-resolution MRI scans were used to extract 2,264 radiomics features from 8 deep brain nuclei, including bilateral thalamus, caudate nucleus, putamen, and globus pallidus. After dimensionality reduction, five independent machine learning classifiers [AdaBoost, Bagging Decision Tree (BDT), Gaussian Process (GP), Logistic Regression (LR), and Random Forest (RF)] were trained on the training set and validated on the test set. Model performance was evaluated using the Area Under the Curve (AUC) metric.

**Results:**

After feature selection, 17 most discriminative radiomics features were retained. Among the models, the BDT-based diagnostic model demonstrated the best performance, achieving AUC values of 1.000 and 0.962 on the training and test sets, respectively. DeLong’s test results indicated that the BDT model significantly outperformed other models. Calibration curve analysis showed that the Parkinson’s disease subtype classification model based on MRI radiomics features exhibited good calibration and stability. Clinical decision curve analysis revealed that the BDT model demonstrated significant clinical net benefits across a wide probability range, indicating high clinical utility.

**Conclusion:**

The BDT model based on MRI radiomics features constructed in this study exhibited excellent performance in differentiating motor subtypes of Parkinson’s disease and can serve as an effective tool for clinical auxiliary diagnosis. This fully automated model is capable of processing MRI data and providing results within 3 min, offering an efficient and reliable solution for the early differentiation of Parkinson’s disease motor subtypes, with significant clinical application value.

## 1 Introduction

Parkinson’s disease (PD) is a complex, progressive neurodegenerative disorder characterized by an insidious onset, high morbidity, and significant disability rates, posing a severe challenge to global public health systems. PD not only threatens the physical and mental health of middle-aged and elderly people but also markedly reduces their quality of life (1). The primary clinical manifestations of PD include motor symptoms (e.g., tremor, rigidity, bradykinesia, and postural instability) and nonmotor symptoms (e.g., sleep disturbances, olfactory dysfunction, autonomic dysfunction, cognitive impairment, and psychiatric disorders). According to the World Health Organization (2), the global number of PD patients exceeded 11.9 million in 2021 and is projected to rise to 25.2 million by 2050, representing a 112% increase. With increasing population ageing, the prevalence of PD is growing at an annual rate of 1.7% (3), imposing a substantial burden on patients, families, and society (4). PD can be classified into two major subtypes on the basis of clinical symptoms: tremor-dominant (TD) and postural instability–gait disturbance (PIGD) (5). The former is characterized by resting tremor (4–6 Hz), rigidity, and bradykinesia, often accompanied by cogwheel rigidity, whereas the latter manifests as balance impairment (positive Romberg sign), freezing of gait, and festination, which are frequently associated with autonomic dysfunction. These subtypes exhibit significant differences in dopaminergic drug responsiveness, disease progression rates, and nonmotor symptom profiles (6, 7). Therefore, developing a rapid and objective diagnostic method to distinguish these subtypes is a critical unmet need for improving patient outcomes.

Currently, PD subtyping relies heavily on subjective clinical assessments, which are prone to cognitive biases and variability in patient cooperation. As a result, clinicians often accurately diagnose PD subtypes only at advanced disease stages (5), delaying targeted treatment. Magnetic resonance imaging (MRI), a noninvasive, radiation-free, high-resolution imaging technique, is widely used for diagnosing and staging neurological disorders. Previous studies have identified structural alterations in the thalamus, caudate nucleus, putamen, and globus pallidus as key factors underlying TD and PIGD subtypes (8, 9). For example, Rosenberg-Katz et al. (10) reported that TD is more closely associated with thalamic damage, whereas PIGD is associated with more severe nigrostriatal degeneration. Dehghan et al. (11) reported widespread grey matter atrophy in PIGD patients, particularly in the frontal lobe, supplementary motor area (SMA), and basal ganglia. Bunzeck et al. (12) demonstrated differences in iron deposition between PD subtypes, with TD patients showing higher basal ganglia iron levels than PIGD patients. However, these studies focused primarily on macroscopic changes and failed to capture subtle structural variations, which limits their diagnostic accuracy and contributes to clinical misclassification. Such misdiagnosis may delay optimal treatment or exacerbate symptoms due to the use of inappropriate medication (13). Moreover, the lack of understanding of microstructural changes hinders the discovery of novel biomarkers.

Radiomics, introduced by Lambin et al. (14), is a computer-aided imaging technique that quantifies subtle, visually imperceptible features in medical images to assist in disease diagnosis and differentiation. It has been widely applied to PD, Alzheimer’s disease (AD), and brain tumours (1, 15, 16). However, existing radiomic models suffer from low accuracy and time-consuming workflows, limiting their clinical adoption (17). Prior to this study, we developed a convolutional neural network (CNN)-based AI model for automated segmentation and measurement of 109 brain regions, completing the process in under 30 s. This study leverages this network to rapidly segment brain nuclei, extract radiomic features, and construct machine learning models to differentiate TD and PIGD.

## 2 Materials and methods

### 2.1 Participants

All PD patients in this study were recruited from the Parkinson’s Progression Markers Initiative (PPMI) database (18). The PPMI patients were newly diagnosed and had not received any medication for at least 6 h. PD diagnosis followed the Movement Disorder Society guidelines. T1-weighted MR images were acquired using a 3 T Siemens scanner with the following parameters: acquisition type = 3D; flip angle = 9.0°; field of view (FOV) = 256 × 256 mm; matrix = 256 × 256; repetition time (TR) = 2300.0 ms; echo time (TE) = 3.0 ms; slice thickness = 1.0 mm; and no slice gap. Exclusion criteria: Patients with head motion >2 mm/° during scans were excluded (*n* = 5). For mild motion, we used N4 bias correction and rigid registration. The study included 135 PD patients (43 PIGD, 92 TD), who were randomly divided into training (80%) and testing (20%) sets.

### 2.2 Brain subregion segmentation

Brain subregion segmentation was performed using a deep learning algorithm based on VB-NET. The preprocessing module performed a series of operations on the MR images for training and testing, including rotation, resampling, resizing, skull stripping, bias field correction, histogram matching, and intensity normalization. All images were standardized to 256 × 256 × 256 mm^3^ in the standard Cartesian LPI coordinate system, with intensity values scaled to (−1, 1). The network training module used an end-to-end deep convolutional neural network, with each sample image and its corresponding brain parcellation atlas as training data. The sample image served as the network input, with the output being the brain parcellation label matching the input image. The network parameters were adjusted on the basis of differences between the output and ground truth parcellations, with training continuing until convergence when the output labels matched the reference parcellations. The network adopted a coarse-to-fine cascaded segmentation strategy, progressively decomposing the complex brain segmentation problem. Upper-level networks provided additional information to enhance lower-level network performance, enabling hierarchical segmentation of large brain regions, medium regions, and fine substructures. The model was trained on 1,800 subjects and achieved a mean Dice coefficient of 0.92 against manual segmentations in 295 test cases. The detailed segmentation methodology is described in our previous publication.

The whole brain was automatically segmented into 109 subregions, including 22 temporal, 20 frontal, 12 parietal, and 8 occipital lobe regions; 8 cingulate and 2 insular subregions; 12 subcortical nuclei; white matter structures; ventricles; cerebellar structures; and other regions (Supplementary material 1). Each patient’s segmentation was completed in under 30 s.

### 2.3 Feature extraction and dimensionality reduction

This study employed the VB-NET deep learning model to automatically quantify the volumes of 109 brain subregions for each patient while simultaneously extracting 2,264 radiomics features from 8 deep brain nuclei (in the bilateral thalamus, caudate nucleus, putamen, and globus pallidus). These features comprehensively captured multidimensional information: 18 first-order statistics and 14 shape features precisely characterized basic morphological properties, reflecting nuclei size proportions and spatial configurations. Texture features were derived from a multimatrix analytical framework, including 21 grey-level cooccurrence matrix (GLCM) features, 16 grey-level run-length matrix (GLRLM) features, 16 grey-level size-zone matrix (GLSZM) features, 5 neighbouring grey-tone difference matrix (NGTDM) features, and 14 grey-level dependence matrix (GLDM) features, enabling in-depth analysis of voxel intensity distribution patterns. For advanced feature construction, we employed a multimodal filtering and frequency-domain transformation strategy to generate hierarchical feature subsets: 24 filters (including the box mean, additive Gaussian noise, binomial blur, etc., and Gaussian filters with *σ* = 0.5, 1, 1.5, 2) were applied for spatial convolution, whereas wavelet transforms extracted 8 directional frequency components (e.g., LLL, LLH) to construct features that integrate spatial and frequency-domain information. All radiomic features underwent z score normalization and complied with the Image Biomarker Standardization Initiative (IBSI) guidelines, with systematic evaluation of feature reproducibility to ensure scientific rigor and cross-cohort applicability.

Feature selection included Relief and least absolute shrinkage and selection operator (LASSO) methods to identify the most robust features. LASSO hyperparameters were optimized via grid search with stratified 5-fold cross-validation on the training set, selecting parameters yielding the highest cross-validated AUC.

### 2.4 Model construction and evaluation

**Figure 1:**
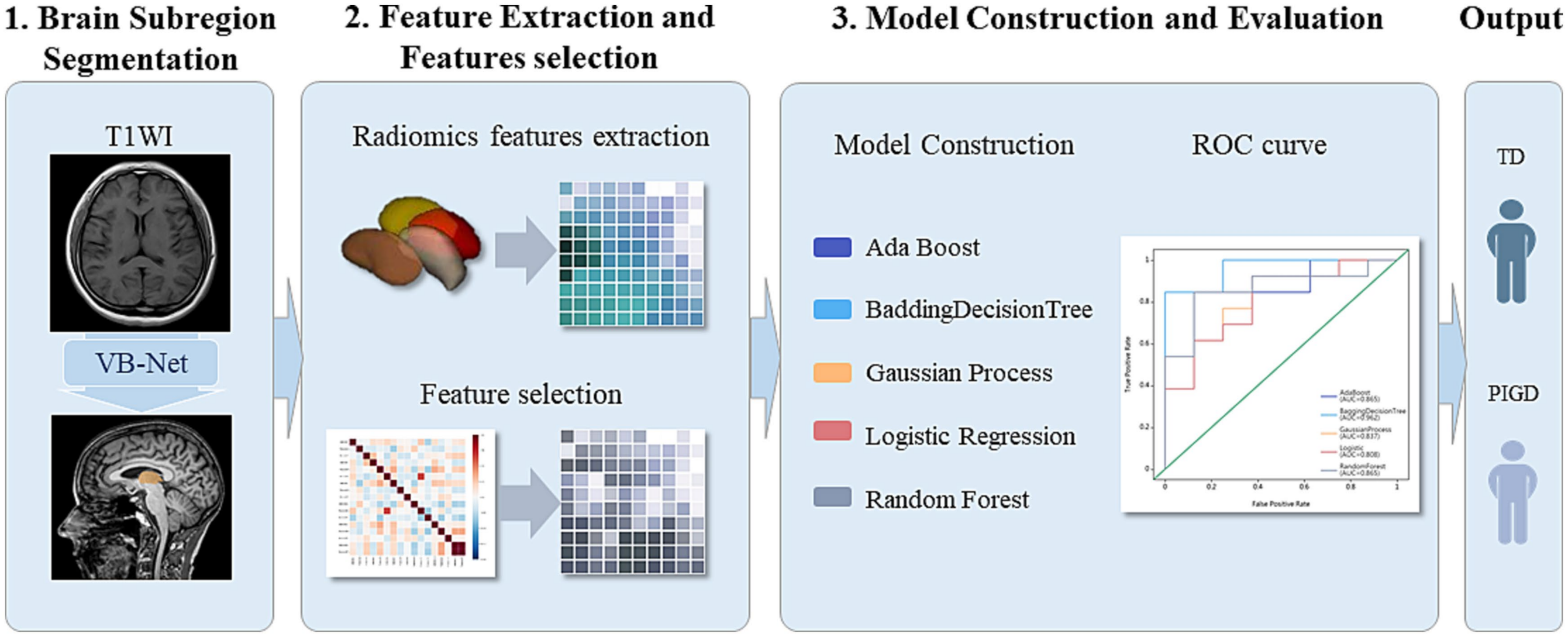
Flowchart of segmentation of deep brain nuclei (bilateral thalamus, caudate nucleus, putamen, and globus pallidus), radiomics feature extraction, and model construction.

Using the selected features, five independent machine learning classifiers (AdaBoost, BDT, GP, LR, RF) were trained on the training set and validated on the test set. Model performance was evaluated in terms of the mean accuracy, sensitivity (recall), specificity, precision with 95% confidence intervals (95% CI), F scores, and area under the receiver operating characteristic curve (AUC). Delong’s test was used to compare the performance between models. Calibration curves were used to assess the agreement between the predicted probabilities and actual outcomes, whereas decision curve analysis (DCA) was used to quantify the clinical net benefit across threshold probabilities to validate the clinical utility (Figure 1).

### 2.5 Statistical analysis

Statistical analyses were performed using R (Version 4.0.4)and Python. For continuous variables, normality was first assessed, followed by Student’s t test for normally distributed data or the Mann–Whitney U test for nonnormally distributed data. Categorical variables were compared using chi-square tests, with two-tailed *p* < 0.05 considered statistically significant. For model comparisons, Delong’s test revealed significant differences in the AUC between classifiers’ ROC curves.

## 3 Results

The study included 135 PD patients (43 PIGD, 92 TD). In the training set, 2,264 radiomics features were extracted from 8 deep brain nuclei. Automated technology precisely quantified the volumes of 109 brain subregions for each patient. For bilateral thalamic regions, the maximum relevance minimum redundancy (MRMR) algorithm initially selected 1,000 representative features from 4,528 radiomic features. Recursive feature elimination cross-validation (RFECV) further refined these to 17 optimal features. Similar feature selection pipelines were applied to the caudate nucleus, putamen, and globus pallidus, yielding region-specific optimal feature sets. Finally, features from all regions were integrated: correlation coefficients first selected 2,195 highly associated features from the full set, MRMR reduced these to 1,000 key features, and RFECV ultimately identified 17 highly discriminative combined features for model construction.

**Figure 2:**
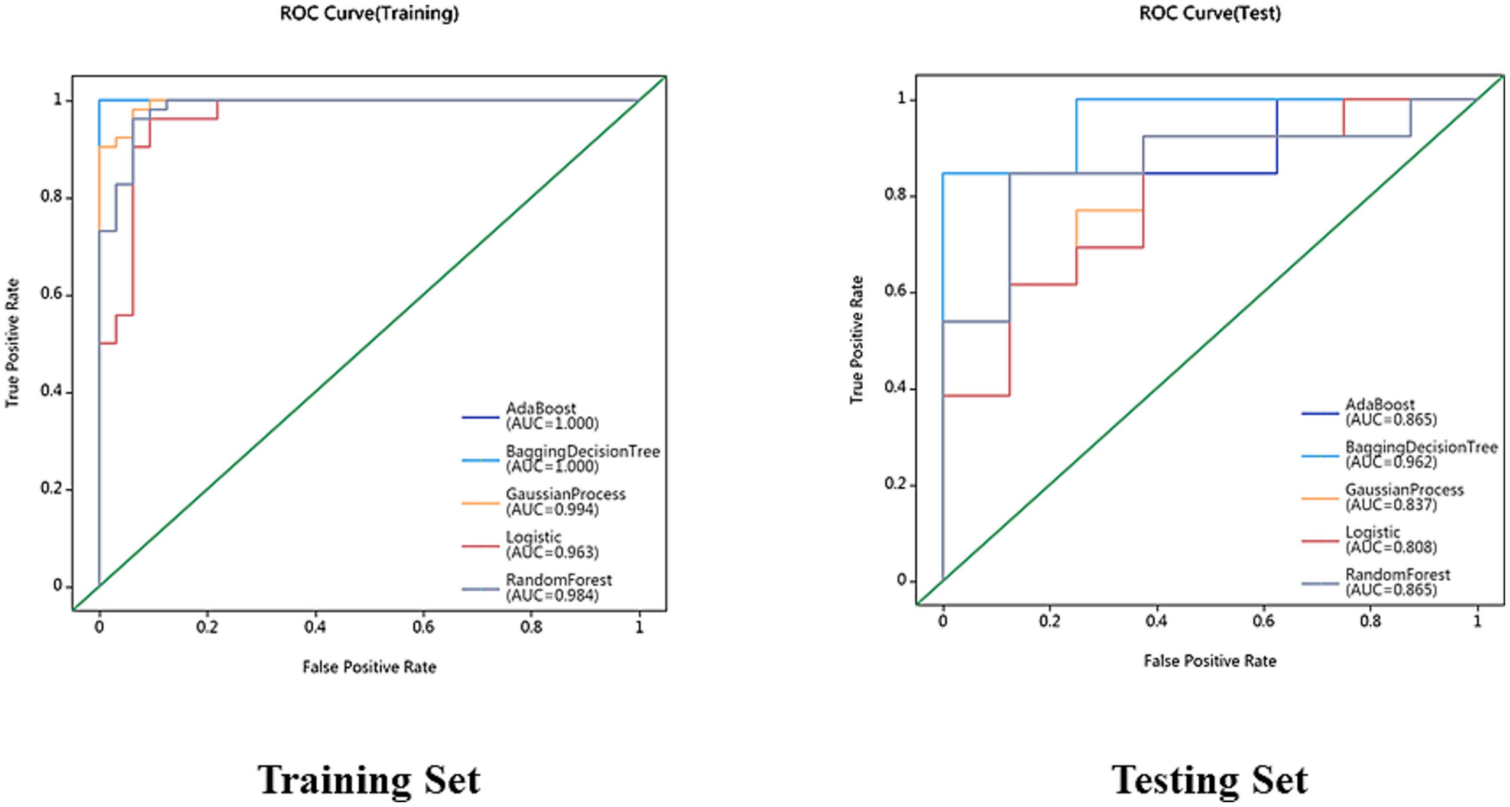
Receiver operating characteristic (ROC) curves for AdaBoost, BDT, GP, LR and RF models in the training set and testing set.

**Figure 3:**
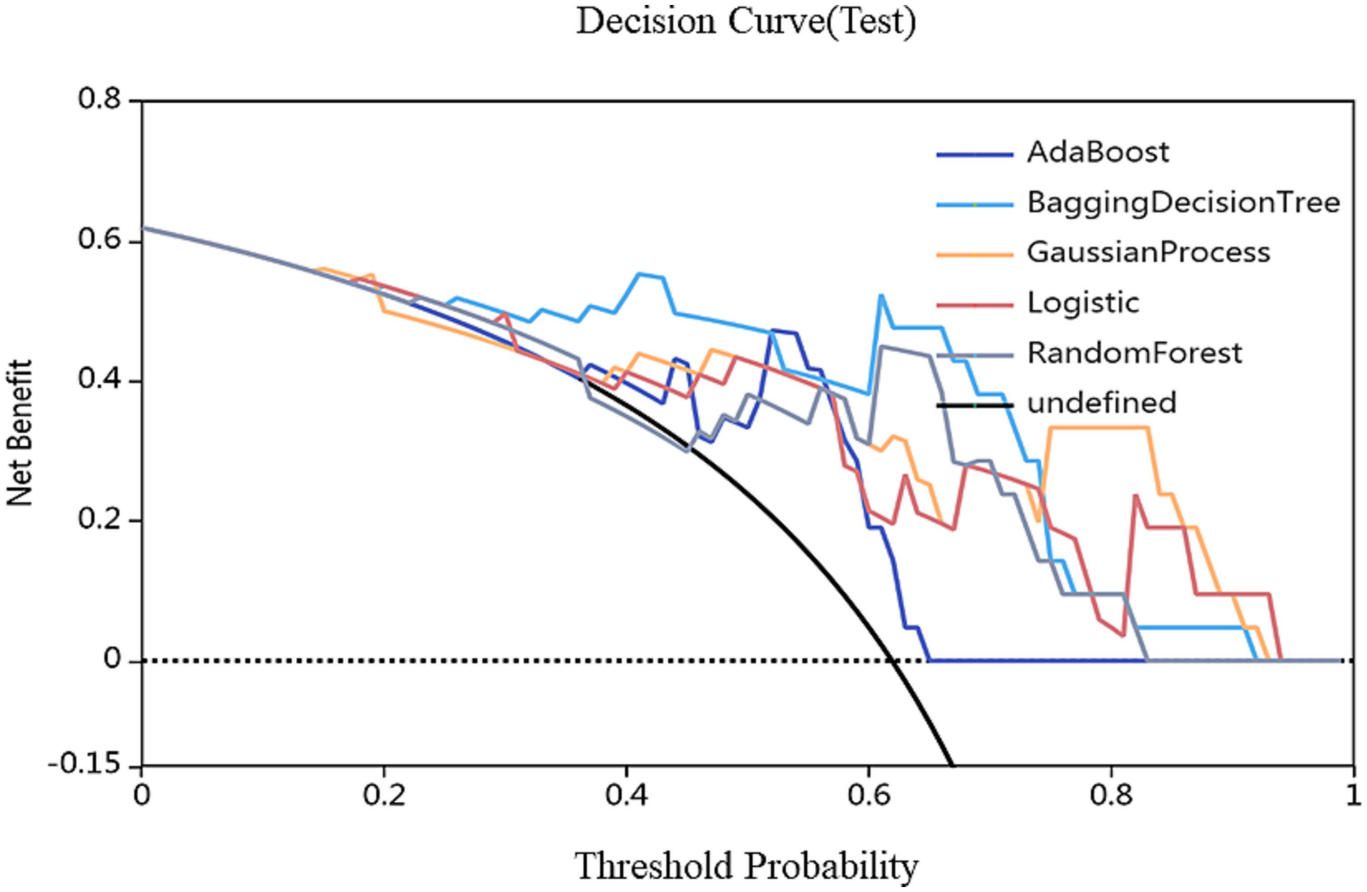
Decision curves of different models. The red, green, and blue lines represent the AdaBoost, BDT, GP, LR and RF models, respectively. The *Y*-axis represents the net benefit, and the *X*-axis represents the threshold probability. Compared with AdaBoost, GP, LR and RF models, BDT with combined features (5 pallidal, 4 putaminal, and 4 caudate features) performed best.

**Figure 4:**
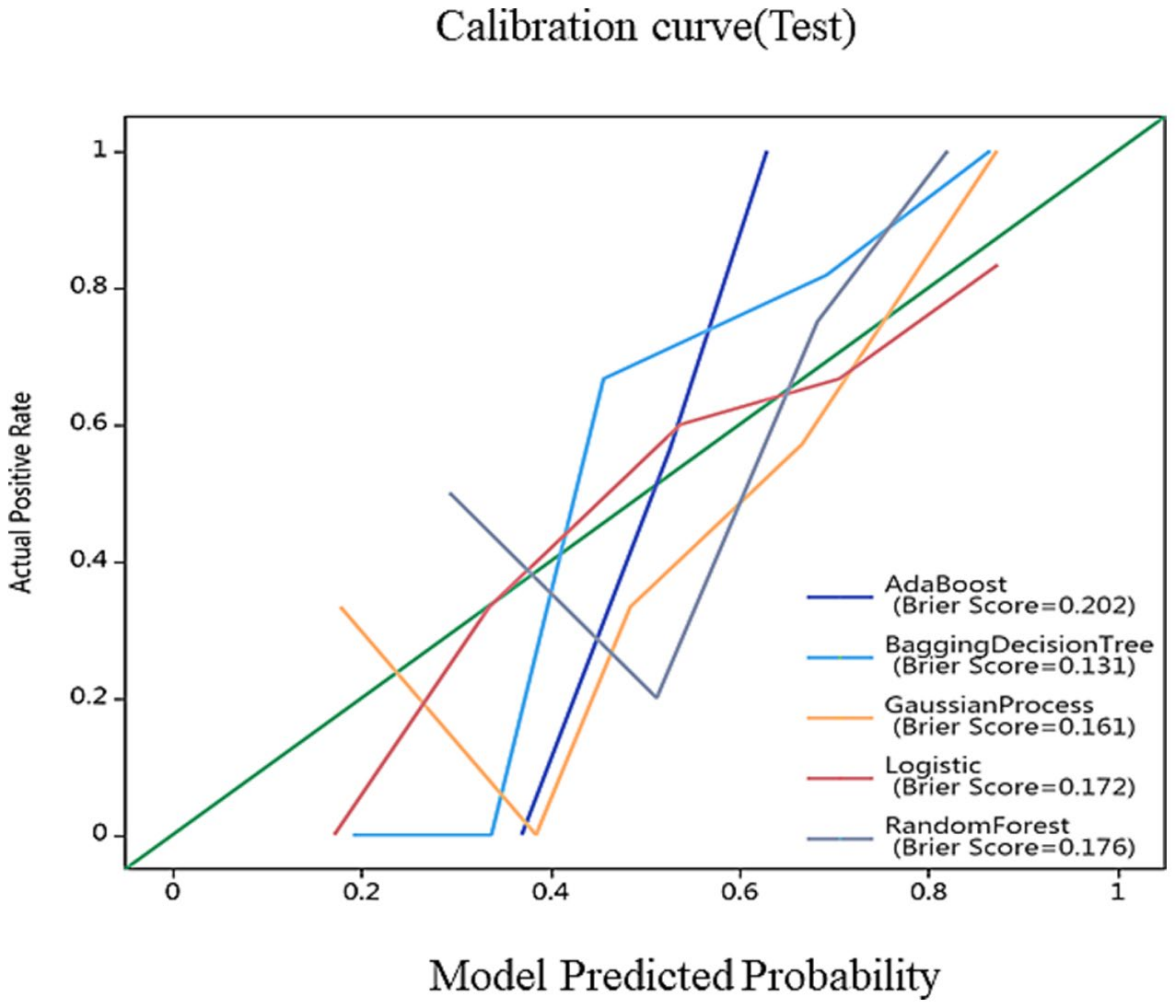
Calibration curves for nomogram goodness of fit. The 45° line represents a perfect match between the actual (*Y*-axis) and predicted (*X*-axis) probabilities of the bar graph. The closer the distance between the two curves, the higher the accuracy.

On the basis of single-region (thalamus, caudate, putamen, globus pallidus) and combined features, we constructed 45 prediction models using five algorithms (AdaBoost, BDT, GP, LR, RF), with detailed performance metrics shown in Table 1. Delong’s test revealed that the BDT model with combined features (5 pallidal, 4 putaminal, and 4 caudate features) performed best, achieving training and testing AUCs of 1.000 and 0.962, respectively (Figure 2), demonstrating excellent classification and generalization capabilities. Calibration curves revealed high agreement between the predicted and actual probabilities (Figure 3), whereas DCA demonstrated significant net clinical benefit across decision thresholds (Figure 4), providing a quantitative tool for personalized treatment decisions (see Table 2).

**Table 1:** Detailed performance indicators of 45 predictive models.

Radiomics features of regions	Models	AUC		Sensitivity		Specificity		Accuracy	
		Training set	Testing set	Training set	Testing set	Training set	Testing set	Training set	Testing set
Caudatum L features	AdaBoost	1	0.5	1	0.923	1	0.375	1	0.714
	BDT	0.999	0.587	1	0.769	0.906	0.25	0.964	0.571
	Gaussian Process	0.885	0.644	0.904	1	0.688	0.375	0.821	0.762
	Logistic	0.944	0.587	0.981	0.846	0.688	0.375	0.869	0.667
	Random Forest	0.867	0.654	0.885	1	0.656	0.5	0.798	0.81
Caudatum R features	AdaBoost	1	0.548	1	0.769	1	0.25	1	0.571
	BDT	0.999	0.606	1	0.615	0.969	0.5	0.988	0.571
	Gaussian Process	0.889	0.481	0.942	0.692	0.656	0.125	0.833	0.476
	Logistic	0.867	0.538	0.942	0.692	0.594	0.125	0.81	0.476
	Random Forest	0.945	0.692	1	0.769	0.531	0.125	0.821	0.524
Pallidum L features	AdaBoost	1	0.577	1	0.769	1	0.375	1	0.619
	BDT	0.996	0.548	1	0.846	0.844	0.375	0.94	0.667
	Gaussian Process	0.893	0.673	0.981	0.846	0.594	0.125	0.833	0.571
	Logistic	0.812	0.673	0.865	0.769	0.5	0.375	0.726	0.619
	Random Forest	0.9	0.702	0.942	0.846	0.688	0.375	0.845	0.667
Pallidum R features	AdaBoost	1	0.779	1	0.769	1	0.625	1	0.714
	BDT	1	0.769	1	0.769	0.844	0.625	0.94	0.714
	Gaussian Process	0.859	0.769	0.904	0.846	0.594	0.625	0.786	0.762
	Logistic	0.834	0.75	0.904	0.846	0.562	0.5	0.774	0.714
	Random Forest	0.936	0.731	0.942	0.923	0.625	0.5	0.821	0.762
Putamen L features	AdaBoost	1	0.76	1	0.846	1	0.5	1	0.714
	BDT	0.993	0.808	1	0.769	0.875	0.5	0.952	0.667
	Gaussian Process	0.858	0.798	0.962	0.846	0.562	0.25	0.81	0.619
	Logistic	0.816	0.76	0.904	0.769	0.5	0.25	0.75	0.571
	Random Forest	0.922	0.885	0.981	0.846	0.688	0.25	0.869	0.619
Putamen R features	AdaBoost	1	0.538	1	0.769	1	0.375	1	0.619
	BDT	0.993	0.481	1	0.538	0.844	0.5	0.94	0.524
	Gaussian Process	0.832	0.577	0.904	0.692	0.594	0.25	0.786	0.524
	Logistic	0.82	0.606	0.865	0.692	0.625	0.375	0.774	0.571
	Random Forest	0.915	0.587	0.981	0.769	0.656	0.375	0.857	0.619
Thalamus L features	AdaBoost	1	0.519	1	0.615	1	0.375	1	0.524
	BDT	0.996	0.538	1	0.846	0.781	0.375	0.917	0.667
	Gaussian Process	0.802	0.625	0.942	0.846	0.406	0.375	0.738	0.667
	Logistic	0.802	0.625	0.885	0.846	0.531	0.375	0.75	0.667
	Random Forest	0.888	0.644	0.981	0.846	0.562	0.375	0.821	0.667
Thalamus R features	AdaBoost	1	0.625	1	0.846	1	0.375	1	0.667
	BDT	0.997	0.635	1	0.769	0.781	0.375	0.917	0.619
	Gaussian Process	0.865	0.644	0.981	0.769	0.562	0.375	0.821	0.619
	Logistic	0.925	0.635	0.962	0.846	0.531	0.375	0.798	0.667
	Random Forest	0.817	0.587	0.885	0.615	0.531	0.375	0.75	0.524
Combined features	AdaBoost	1	0.865	1	0.846	1	0.5	1	0.714
	BDT	1	0.962	1	0.923	0.969	0.75	0.988	0.857
	Gaussian Process	0.994	0.837	1	0.923	0.906	0.625	0.964	0.81
	Logistic	0.963	0.808	0.962	0.923	0.906	0.625	0.94	0.81
	Random Forest	0.984	0.865	1	0.923	0.812	0.5	0.929	0.762

BDT, Bagging Decision Tree; AUC, Area Under Curve.

**Table 2:** Details of 17 selected features.

Feature name	Filter type	Feature class	Biological interpretation	IBSI compliant	Rank
Caudate_R_log_glszm_log-sigma-2-0-mm-3D-GrayLevelNonUniformity	Laplacian of Gaussian (σ = 2)	GLSZM	Heterogeneity of gray-level zones	Yes	1
Caudate_L_additivegaussiannoise_firstorder_Skewness	Additive Gaussian Noise	First-order	Intensity distribution asymmetry	Yes	1
Putamen_L_additivegaussiannoise_glrlm_ShortRunHighGrayLevelEmphasis	Additive Gaussian Noise	GLRLM	Presence of short bright runs (iron deposition?)	Yes	1
Caudate_L_boxsigmaimage_gldm_DependenceNonUniformityNormalized	Box Sigma	GLDM	Spatial consistency of gray-level dependencies	Yes	1
Caudate_L_boxsigmaimage_gldm_DependenceVariance	Box Sigma	GLDM	Variability in gray-level dependencies	Yes	1
Pallidum_R_boxmean_gldm_SmallDependenceLowGrayLevelEmphasis	Box Mean	GLDM	Small dark regions (possible microstructural gaps)	Yes	1
Caudate_R_boxmean_glszm_GrayLevelNonUniformity	Box Mean	GLSZM	Non-uniformity of gray-level zones	Yes	1
Thalamus_R_boxsigmaimage_glszm_SmallAreaEmphasis	Box Sigma	GLSZM	Prevalence of small areas (e.g., gliosis)	Yes	1
Pallidum_L_boxsigmaimage_glszm_SmallAreaEmphasis	Box Sigma	GLSZM	Small lesion-like structures	Yes	1
Pallidum_L_boxsigmaimage_glrlm_LongRunHighGrayLevelEmphasis	Box Sigma	GLRLM	Elongated bright regions (axonal integrity?)	Yes	1
Pallidum_L_log_firstorder_log-sigma-4-0-mm-3D-Skewness	Wavelet (LLL)	First-order	Intensity distribution tail direction	Yes	1
Caudate_R_boxsigmaimage_gldm_DependenceNonUniformityNormalized	Box Sigma	GLDM	Repeats spatial dependency measure	Yes	2
Pallidum_R_log_glrlm_log-sigma-4-0-mm-3D-ShortRunHighGrayLevelEmphasis	Wavelet (LLH)	GLRLM	Focal bright spots (iron clusters?)	Yes	3
Caudate_L_log_ngtdm_log-sigma-2-0-mm-3D-Contrast	Wavelet (LHL)	NGTDM	Local intensity variation	Yes	4
Caudate_L_log_glszm_log-sigma-2-0-mm-3D-GrayLevelNonUniformity	Wavelet (LHH)	GLSZM	Repeats heterogeneity measure	Yes	5
Putamen_L_additivegaussiannoise_glcm_Autocorrelation	Additive Gaussian Noise	GLCM	Pattern repetition (dopaminergic terminal density)	Yes	6
Pallidum_L_log_glrlm_log-sigma-4-0-mm-3D-ShortRunLowGrayLevelEmphasis	Wavelet (HLL)	GLRLM	Short dark runs (possible neuronal loss)	Yes	7

## 4 Discussion

TD and PIGD differ not only in motor symptoms (resting tremor vs. gait freezing) but also in nonmotor symptom patterns (e.g., sleep disturbances, autonomic dysfunction) (19), leading to varied responses to PD medications (e.g., dopamine agonists vs. levodopa) (20, 21) and novel therapies such as transcranial magnetic stimulation. However, current PD subtyping relies on clinical differentiation, which is often delayed until intermediate or advanced stages (18, 22), resulting in late intervention. Thus, establishing early, rapid, and accurate subtyping methods could optimize symptom management and enable timely targeted interventions.

This study implemented automated whole-brain subregion segmentation using VB-NET, a U-NET variant. As an efficient medical image segmentation model, VB-NET automatically learns spatial features and boundary information of brain subregions without manual annotation, reducing the processing time from hours to seconds while improving precision and consistency. Previous segmentation methods have relied on manual delineation or simple algorithms. For example, Jingnan et al. (23) manually outlined the caudate and putamen to calculate the striatal-to-occipital uptake ratios (SORs) of 18F-DOPA in PD patients versus controls. These manual approaches are time-consuming, subjective, and inconsistent. Crucially, our model uses routine 3D-T1WI without advanced functional imaging, significantly reducing costs and enhancing clinical accessibility. Furthermore, VB-NET automatically extracted 2,264 radiomics features from key nuclei (the caudate, putamen, globus pallidus, and thalamus), capturing multidimensional morphological and textural information. Five machine learning algorithms (AB, BDT, GP, LR, and RF) were employed for automated TD/PIGD classification and were validated on independent test sets. The BDT classifier achieved exceptional performance (training AUC = 1.000; testing AUC = 0.962), demonstrating high accuracy and generalizability. Compared with prior studies, this work represents the first fully automated TD/PIGD classification pipeline, eliminating subjective clinical assessments and completing diagnosis within 3 min—offering clinicians a rapid, objective tool for personalized treatment planning.

Feature selection identified 17 core radiomic features with distinct regional distributions: caudate (8), globus pallidus (6), putamen (2), and thalamus (1). This pattern aligns with PD pathophysiology: pallidal (6) and putaminal (2) texture features showed early pathological (24) sensitivity, with abnormalities detectable in preclinical stages (25) (e.g., REM sleep behaviour disorder), preceding macroscopic atrophy. TD was strongly correlated with putaminal texture features (26) (e.g., autocorrelation). As a key node in the direct pathway, putamen dysfunction directly contributes to classic PD motor symptoms (tremor, movement initiation difficulty) (27). There exist distinct patterns of iron deposition in the basal ganglia between Parkinson’s disease (PD) patients with postural instability/gait disorder (PIGD) and those with tremor dominance (TD). Compared with the TD group, the PIGD group exhibits significantly higher susceptibility values in the left putamen (PUT), particularly in patients whose major symptomatic side is the right limb. This indicates that the distribution of iron deposition in the basal ganglia is more extensive in the PIGD group, involving not only the substantia nigra (SN) but also regions such as the caudate nucleus (CN) and putamen, with a more severe degree of iron deposition in the putamen (28). From the perspective of the correlation between iron deposition and symptoms, the damaged putamen is likely to be the pathophysiological basis for gait and posture disorders in Parkinson’s disease. Reduced autocorrelation indicates disrupted textural periodicity, which is consistent with PET evidence of decreased dopamine receptor density (29), reflecting early synaptic degeneration. PIGD involved primarily pallidal (5) and thalamic (1) features. The globus pallidus, a core indirect pathway node, exhibits degeneration-related motor inhibition (rigidity, bradykinesia) (30). Changes in texture homogeneity may reflect iron deposition (T2*/SWI hypointensity) or neuronal loss (31), whereas the presence of thalamic abnormalities suggest upstream motor circuit disruption (32). GrayLevelNonUniformity frequently appeared in the bilateral caudate (Caudate_R, Caudate_L, Caudate_R_boxmean), indicating microstructural heterogeneity from neuronal loss, iron deposition, or uneven Lewy body distribution. SmallAreaEmphasis in the right thalamus (Thalamus_R) and left globus pallidus (Pallidum_L) suggested early microstructural changes (e.g., enlarged perivascular spaces, focal gliosis), matching histopathological “nonuniform degeneration” patterns. These features not only enabled high-accuracy classification but also revealed spatiotemporal patterns of basal ganglia involvement in PD subtypes—early putamen/pallidum texture changes driving motor subtype differentiation, with thalamic features contributing to circuit-level dysfunction. Thus, these radiomic features provide quantifiable imaging biomarkers for precise subtyping and pathological investigation.

Our study had several limitations. This study had a retrospective cross-sectional design. Future prospective longitudinal studies should examine whether feature changes predict disease conversion (e.g., PD to dementia). The PD sample size was relatively small. All the data were acquired on 3 T Siemens scanners, but model generalizability requires validation across multicentre datasets. Future studies need to further verify the stability and universality of the conclusions of this study by expanding the sample size (including multi center and multi-regional data) and conducting independent external cohort validation. The current research results cannot directly verify their predictive efficacy for subtype progression trajectories (such as the rate of motor symptom deterioration, patterns of cognitive decline, etc.). In the future, we will conduct a longitudinal follow-up study based on a multi-center cohort to verify the longitudinal stability and clinical practical value of potential predictive biomarkers. The sole focus of this study was imaging features. Future work should integrate clinical scores (UPDRS, MMSE) into diagnostic tools. Although radiomic features demonstrated high accuracy in PD subtyping, their underlying pathological basis requires further elucidation.

## 5 Conclusion

We developed an AI model that accurately differentiates PD subtypes within 3 min using routine MRI data. This fully automated approach significantly improves diagnostic efficiency and holds substantial clinical potential for precise PD subtyping.

## Data Availability

Publicly available datasets were analyzed in this study. This data can be found at: Parkinson’s Progression Markers Initiative (PPMI) database https://www.ppmi-info.org/.
