# Supplementary material for: Automatic differentiation of Parkinson’s disease motor subtypes based on deep learning and radiomics
Source: Front Neurol. 2025 Sep 4;16:1650985. doi: 10.3389/fneur.2025.1650985 (PMC12445147; doi:10.3389/fneur.2025.1650985)
Supplement: Supplementary file 1 [file Table_1.DOCX]

**Supplementary Material 1:**

| Region | DK109 |
| --- | --- |
| 1 | Precentral_L |
| 2 | Precentral_R |
| 3 | Postcentral_L |
| 4 | Postcentral_R |
| 5 | Paracentral_L |
| 6 | Paracentral_R |
| 7 | Frontal_Sup_L |
| 8 | Frontal_Sup_R |
| 9 | Frontal_Mid_Rostral_L |
| 10 | Frontal_Mid_Rostral_R |
| 11 | Frontal_Mid_Caudal_L |
| 12 | Frontal_Mid_Caudal_R |
| 13 | Frontalpole_L |
| 14 | Frontalpole_R |
| 15 | Orbitofrontal_Lat_L |
| 16 | Orbitofrontal_Lat_R |
| 17 | Orbitofrontal_Med_L |
| 18 | Orbitofrontal_Med_R |
| 19 | Parsopercularis_L |
| 20 | Parsopercularis_R |
| 21 | Parsorbitalis_L |
| 22 | Parsorbitalis_R |
| 23 | Parstriangularis_L |
| 24 | Parstriangularis_R |
| 25 | Insula_L |
| 26 | Insula_R |
| 27 | Cingulum_Ant_L |
| 28 | Cingulum_Ant_R |
| 29 | Cingulum_Mid_L |
| 30 | Cingulum_Mid_R |
| 31 | Cingulum_Post_L |
| 32 | Cingulum_Post_R |
| 33 | Isthmuscingulate_L |
| 34 | Isthmuscingulate_R |
| 35 | Hippocampus_L |
| 36 | Hippocampus_R |
| 37 | Parahippocampal_L |
| 38 | Parahippocampal_R |
| 39 | Amygdala_L |
| 40 | Amygdala_R |
| 41 | Caudate_L |
| 42 | Caudate_R |
| 43 | Putamen_L |
| 44 | Putamen_R |
| 45 | Pallidum_L |
| 46 | Pallidum_R |
| 47 | Thalamus_L |
| 48 | Thalamus_R |
| 49 | Accumbens_Area_L |
| 50 | Accumbens_Area_R |
| 51 | VentralDC_L |
| 52 | VentralDC_R |
| 53 | Choroid_Plexus_L |
| 54 | Choroid_Plexus_R |
| 55 | Ventricle_Lat_L |
| 56 | Ventricle_Lat_R |
| 57 | Ventricle_Inf_Lat_L |
| 58 | Ventricle_Inf_Lat_R |
| 59 | Parietal_Sup_L |
| 60 | Parietal_Sup_R |
| 61 | Parietal_Inf_L |
| 62 | Parietal_Inf_R |
| 63 | Cuneus_L |
| 64 | Cuneus_R |
| 65 | Entorhinal_L |
| 66 | Entorhinal_R |
| 67 | Fusiform_L |
| 68 | Fusiform_R |
| 69 | Lingual_L |
| 70 | Lingual_R |
| 71 | Pericalcarine_L |
| 72 | Pericalcarine_R |
| 73 | Precuneus_L |
| 74 | Precuneus_R |
| 75 | Supramarginal_L |
| 76 | Supramarginal_R |
| 77 | Temporal_Sup_L |
| 78 | Temporal_Sup_R |
| 79 | Temporal_Mid_L |
| 80 | Temporal_Mid_R |
| 81 | Temporal_Inf_L |
| 82 | Temporal_Inf_R |
| 83 | Temporalpole_L |
| 84 | Temporalpole_R |
| 85 | Temporal_Sup_Banks_L |
| 86 | Temporal_Sup_Banks_R |
| 87 | Transversetemporal_L |
| 88 | Transversetemporal_R |
| 89 | Occipital_Lat_L |
| 90 | Occipital_Lat_R |
| 91 | Cerebral_WM_L |
| 92 | Cerebral_WM_R |
| 93 | Cerebellum_Cortex_L |
| 94 | Cerebellum_Cortex_R |
| 95 | Cerebellum_WM_L |
| 96 | Cerebellum_WM_R |
| 97 | Ventricle_3rd |
| 98 | Ventricle_4th |
| 99 | Pons |
| 100 | CSF |
| 101 | Optic_Chiasm |
| 102 | CC_Anterior |
| 103 | CC_Mid_Anterior |
| 104 | CC_Central |
| 105 | CC_Mid_Posterior |
| 106 | CC_Posterior |
| 107 | Midbrain |
| 108 | Medulla |
| 109 | SCP |
